# Association Between Participant Satisfaction and Self‐Reported Clinical Practice Behaviors in a Nationwide Guideline Education Program

**DOI:** 10.1002/npr2.70147

**Published:** 2026-07-14

**Authors:** Kazuhiko Yamamuro, Kenta Ide, Yusuke Arai, Hiroyuki Muraoka, Toru Horinouchi, Yasushi Kawamata, Keisuke Mori, Shinichiro Ochi, Takashi Tsuboi, Hirotaka Yamagata, Hitoshi Iida, Yuka Yasuda, Junya Matsumoto, Naoki Hashimoto, Kazutaka Ohi, Toshiaki Onitsuka, Hisashi Yamada, Shusuke Numata, Hikaru Hori, Ken Inada, Koichiro Watanabe, Norio Yasui‐Furukori, Ryota Hashimoto

**Affiliations:** ^1^ Center for Health Control, Nara Medical University Kashihara Nara Japan; ^2^ Department of Psychiatry Nara Medical University, School of Medicine Kashihara Nara Japan; ^3^ Department of Hospital Pharmacy Hospital of University of Occupational and Environmental Health Kitakyushu Fukuoka Japan; ^4^ Department of Pathology of Mental Diseases National Institute of Mental Health, National Center of Neurology and Psychiatry Kodaira Tokyo Japan; ^5^ Department of Community Mental Health Shinshu University School of Medicine, Matsumoto Nagano Japan; ^6^ Department of Psychiatry Kitasato University School of Medicine Sagamihara Kanagawa Japan; ^7^ Department of Psychiatry Hokkaido University Graduate School of Medicine Sapporo Hokkaido Japan; ^8^ Department of Psychiatry Dokkyo Medical University School of Medicine Mibu Tochigi Japan; ^9^ Department of Psychiatry The Jikei University School of Medicine Minato‐ku Tokyo Japan; ^10^ Department of Neuropsychiatry Molecule and Function, Ehime University Graduate School of Medicine Toon Ehime Japan; ^11^ Department of Neuropsychiatry Kyorin University School of Medicine Mitaka Tokyo Japan; ^12^ Department of Psychiatry Faculty of Medicine, Fukuoka University Fukuoka Fukuoka Japan; ^13^ Department of Psychiatry Gifu University Graduate School of Medicine Gifu Gifu Japan; ^14^ NHO Sakakibara National Hospital Tsu Mie Japan; ^15^ Department of Neuropsychiatry Hyogo Medical University Nishinomiya Hyogo Japan; ^16^ Department of Psychiatry Graduate School of Biomedical Science, Tokushima University Tokushima Tokushima Japan

**Keywords:** major depressive disorder, personal satisfaction, psychiatrists, schizophrenia, self‐report

## Abstract

**Background:**

Educational interventions are widely used to promote guideline‐concordant psychiatric practice. However, it remains unclear whether participants' subjective satisfaction translates into actual changes in clinical behavior (CB). This study examined the association between subjective assessment (SA) scores of the EGUIDE training programs for schizophrenia and major depressive disorder (MDD) and CB.

**Methods:**

In this multicenter observational study, we analyzed data from psychiatrists who participated in the EGUIDE training program. SA scores were obtained immediately after training using standardized questionnaires assessing satisfaction with program content, knowledge, skills, and future clinical intentions. CB was evaluated using self‐report measures of guideline‐concordant practice in general, schizophrenia‐specific, and MDD‐specific domains. Associations between SA and CB scores were examined using Spearman's rank correlation coefficients.

**Results:**

A total of 1399 psychiatrists were included in the analysis. The comprehensive SA score showed a significant positive correlation with the comprehensive CB score (*r* = 0.19, *p* < 0.001). Likewise, schizophrenia‐ and MDD‐specific SA scores were positively correlated with all CB domains, including general guideline use and disorder‐specific practices (*ρ* = 0.14–0.19, all *p* < 0.001). Although effect sizes were small, the associations were consistent across disorders and clinical domains.

**Conclusions:**

Higher satisfaction with guideline‐based educational programs was associated with greater self‐reported guideline‐concordant CBs, suggesting that positive educational experiences may support improvements in psychiatric practice at the national level.

## Abstract 2

Higher satisfaction with nationwide guideline education programs was associated with greater self‐reported guideline‐concordant clinical behaviors among psychiatrists in Japan. These findings suggest that positive educational experiences may support implementation of evidence‐based psychiatric practice.

## Graphical abstract

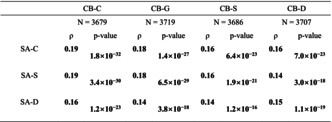

## 1 Introduction

Treatment guidelines for schizophrenia (SZ) and major depressive disorder (MDD) provide evidence‐based standards of care and play a crucial role in improving the quality of medical practice. However, the mere publication of guidelines does not ensure their sufficient acceptance in routine clinical practice, and deviations from the recommended treatments, such as non‐guideline‐concordant prescriptions and management, have been reported across many medical fields [1, 2]. In psychiatry, treatment practices by physicians that are inconsistent with guideline recommendations, including antipsychotic polypharmacy, excessive dosing, and long‐term prescription of benzodiazepines, have been documented worldwide [3, 4, 5] and repeatedly reported in Japan [6, 7, 8, 9, 10, 11, 12, 13, 14, 15, 16, 17].

Against this background, the Effectiveness of Guidelines for Dissemination and Education in Psychiatric Treatment (EGUIDE) project was launched in Japan in 2016 [18, 19]. This nationwide initiative conducts educational programs for psychiatrists at medical institutions across Japan to promote physicians' understanding and implementation of clinical practice guidelines for patients with SZ and MDD. The effectiveness of the program has been demonstrated by improvements in participating physicians' knowledge test scores assessed before and after training sessions [20, 21]. In the post‐training subjective assessment (SA) scores, > 90% of physicians highly rated the program in five of the six evaluated domains: content, recommendations, knowledge, skills, and adherence [22]. In addition, improvements in physicians' self‐reported clinical behavior (CB) scores assessed via practice questionnaires were sustained for up to 8 years following participation in the program [23, 24]. Furthermore, quality indicators evaluating physicians' prescription patterns and treatment behaviors at institutions that participated in the EGUIDE program have been shown to improve gradually over time [18, 19], and these indicators were also provided to each institution as objective feedback.

Within the EGUIDE framework, higher SA scores may increase physicians' intentions to practice in accordance with clinical guidelines, which may be reflected in their CB and contribute to the optimization of treatment practices. We have previously shown that self‐reported CB is associated with quality indicators, supporting CB as a proxy for physicians' guideline‐concordant intentions [25]. However, to our knowledge, no nationwide study has systematically examined the relationship between physicians' SA scores and CB. Therefore, in this study, we aimed to investigate the association between SA scores from the EGUIDE training programs for SZ and MDD and CB among EGUIDE participants.

## 2 Methods

### 2.1 Study Design and Participants

In this multicenter observational study, we analyzed data from psychiatrists across Japan who voluntarily participated in the EGUIDE project. Written informed consent was obtained from all participants. SA was conducted after completion of the EGUIDE training program, and clinical practice behaviors were assessed using practice questionnaires. The study was approved by the Ethics Committee of the National Center of Neurology and Psychiatry (approval no. B2022‐004), with additional approval from participating institutions. It was registered with the University Hospital Medical Information Network (UMIN000022645) and conducted in accordance with the Declaration of Helsinki.

### 2.2 Subjective Assessment

The SA scores for each guideline‐based training program were evaluated using a self‐report questionnaire completed immediately after the training sessions [22]. The questionnaire comprised six items rated on a 5‐point Likert scale (1 = very dissatisfied to 5 = very satisfied). Total scores (maximum = 30) were converted to a 0%–100% scale and defined as SA scores for the SZ (SA‐S) and MDD (SA‐D) programs. The comprehensive SA score (SA‐C) was calculated as the mean of SA‐S and SA‐D. Questionnaire items are listed in Table S2.

### 2.3 Clinical Behavior

The CB scores were assessed using a self‐report questionnaire administered annually after participation in the training program to subjectively evaluate the degree of guideline adherence [23, 24]. This self‐assessment questionnaire assessed physicians' self‐perceived CBs. Items were rated on a 5‐point Likert scale reflecting implementation frequency (0%–100%), with representative scores of 10%, 30%, 50%, 70%, and 90%. Responses indicating no opportunity to implement were excluded, and mean scores were calculated. CB scores were derived for three domains: general guideline use (CB‐G), SZ‐related behaviors (CB‐S), and MDD‐related behaviors (CB‐D). The comprehensive CB score (CB‐C) was defined as the mean of these three domain scores. Questionnaire items are provided in Table S3.

### 2.4 Statistical Analysis

All analyses were performed using IBM SPSS Statistics for Windows, Version 26.0 (IBM Corp., Armonk, NY, USA). Analyses were conducted among physicians with complete data for both SA and CB. CB questionnaires were administered annually, and some physicians contributed CB data in multiple years. Correlation analyses were performed using Spearman's rank correlation coefficient to examine the association between the SA and CB scores. To account for multiple comparisons, a Bonferroni correction was applied, and the threshold for statistical significance was set at *p* < 0.0056 (0.05/9).

## 3 Results

### 3.1 Demographic Data

Of the 1399 participants, 1001 (71.6%) were men. The mean age of the participants was 32.6 years, and the mean duration of clinical experience in psychiatry was 3.7 years. Regarding SA, the mean SA‐C, SA‐S, and SA‐D scores were 83.8, 83.4, and 83.9, respectively. Regarding CB, the mean CB‐C score was 63.9. The mean scores for CB‐G, CB‐S, and CB‐D were 59.6, 66.0, and 66.1, respectively (Table 1).

**TABLE 1:** Demographic characteristics of participating psychiatrists.

	Mean	SD
	*N* = 1399	
Age, y	32.6	7.0
Sex, *n* (%)		
Male	1001 (71.6)	
Female	398 (28.4)	
Psychiatric history, y	3.7	5.7
SA‐C	83.8	7.2
SA‐S	83.4	7.6
SA‐D	83.9	7.9
CB‐C	63.9	13.5
CB‐G	59.6	19.0
CB‐S	66.0	13.1
CB‐D	66.1	14.3

Abbreviations: CB‐C, comprehensive clinical behavior score; CB‐D, clinical behavior score for depression‐related practices; CB‐G, clinical behavior score for general guideline use; CB‐S, clinical behavior score for schizophrenia‐related practices; SA‐C, comprehensive satisfaction assessment; SA‐D, satisfaction assessment for the depression program; SA‐S, satisfaction assessment for the schizophrenia program; SD, standard deviation.

### 3.2 Association Between SA and CB Scores

The associations between the SA and CB scores are summarized in Table 2. The SA‐C score was significantly positively correlated with the CB‐C (*ρ* = 0.19, *p* = 1.8 × 10^−32^), CB‐G (*ρ* = 0.18, *p* = 1.4 × 10^−27^), CB‐S (*ρ* = 0.16, *p* = 6.4 × 10^−23^), and CB‐D (*ρ* = 0.16, *p* = 7.0 × 10^−23^) scores. Similarly, the SA‐S score was positively correlated with the CB‐C (*ρ* = 0.19, *p* = 3.4 × 10^−30^), CB‐G (*ρ* = 0.18, *p* = 6.5 × 10^−29^), CB‐S (*ρ* = 0.16, *p* = 1.9 × 10^−21^), and CB‐D (*ρ* = 0.14, *p* = 3.0 × 10^−18^) scores. In addition, the SA‐D was also positively correlated with CB‐C (*r* = 0.16, *p* = 1.2 × 10^−23^), CB‐G (*ρ* = 0.14, *p* = 3.8 × 10^−18^), CB‐S (*ρ* = 0.14, p = 1.2 × 10^−16^), and CB‐D (ρ = 0.15, *p* = 1.1 × 10^−19^) scores. Collectively, all SA scores were positively associated with multiple domains of CB.

**TABLE 2:** Correlations between overall implementation and satisfaction rates.

	CB‐C		CB‐G		CB‐S		CB‐D	
	*N* = 3679		*N* = 3719		*N* = 3686		*N* = 3707	
	*ρ*	*p*	*ρ*	*p*	*ρ*	*p*	*ρ*	*p*
SA‐C	**0.19**	**1.8** × **10** ^**−32**^	**0.18**	**1.4** × **10** ^**−27**^	**0.16**	**6.4** × **10** ^**−23**^	**0.16**	**7.0** × **10** ^**−23**^
SA‐S	**0.19**	**3.4** × **10** ^**−30**^	**0.18**	**6.5** × **10** ^**−29**^	**0.16**	**1.9** × **10** ^**−21**^	**0.14**	**3.0** × **10** ^**−18**^
SA‐D	**0.16**	**1.2** × **10** ^**−23**^	**0.14**	**3.8** × **10** ^**−18**^	**0.14**	**1.2** × **10** ^**−16**^	**0.15**	**1.1** × **10** ^**−19**^

*Note:* Correlation coefficients (*ρ*) were calculated using Spearman's rank correlation. All analyses were performed on complete cases. Boldface indicates the largest correlation coefficients in each row. A Bonferroni‐corrected significance threshold was applied (0.05/9 = 0.0056).

Abbreviations: CB‐C, comprehensive clinical behavior score; CB‐D, clinical behavior score for depression‐related practices; CB‐G, clinical behavior score for general guideline use; CB‐S, clinical behavior score for schizophrenia‐related practices; SA‐C, comprehensive satisfaction assessment; SA‐D, satisfaction assessment for the depression program SA‐S, satisfaction assessment for the schizophrenia program.

## 4 Discussion

In this study, all SA scores from the EGUIDE training programs were significantly positively correlated with all CB domains. Although the correlations were modest (*r* = 0.14–.19), this consistent pattern was observed across both SZ and MDD programs. Moreover, treatment behaviors showed similar temporal improvement trajectories across disorders, suggesting reproducible effects of the training programs [18, 19]. The finding that disorder‐specific SA scores were also associated with CB scores in other domains indicates that the SA scores of a particular training program may influence overall clinical judgment and practice beyond the target disorder. In medical education, the generalization of learning experiences to other clinical contexts is considered essential [26]. A positive evaluation of training content may broadly reinforce guideline‐based attitudes and decision making.

Although the correlation coefficients were modest, their magnitude was consistent with that reported in previous studies, reflecting the multifactorial nature of satisfaction and CB change [27, 28]. Even when educational interventions improve behavior at the group level, the correspondence between SA scores and behavioral change at the individual level is often weak [29]. Within the EGUIDE project, CB scores have been shown to increase over the long term following training [23, 24]. The weak correlations observed may also be partly attributable to a ceiling effect, as SA scores were clustered at the higher end of the scale (mean≈84, SD≈7), consistent with prior EGUIDE studies [22]. Nevertheless, despite this restricted range, SA scores remained significantly associated with CB, suggesting that high‐quality training and favorable subjective evaluations may be linked to improved clinical practice.

The strengths of this study include the use of a large‐scale multicenter sample and systematic examination of the association between SA and CB scores. However, this study has several limitations. First, because this was a cross‐sectional study, the directionality and causality of the observed associations could not be determined. Second, the CB measures were based on self‐reported assessments and may therefore have been influenced by response bias or social desirability bias. Third, although the observed effect sizes were modest, behavioral changes after educational interventions are generally multifactorial, and even small but consistent associations may still be clinically meaningful in large‐scale implementation programs. Finally, the study sample predominantly comprised younger psychiatrists with relatively short clinical experience, which may limit the generalizability of the findings to more experienced clinicians.

In this study, the SA of EGUIDE training lectures was associated with CB, suggesting that positive evaluations may reflect clinicians' engagement in guideline‐concordant practices. Improving participant satisfaction alone may not be sufficient to change clinical practice. Rather, satisfaction may serve as an indicator of engagement within a broader, multifaceted implementation strategy.

## Author Contributions

K.Y. and R.H. were critically involved in collecting and analyzing the data and wrote the first draft of the manuscript. K.Y., Kenta I, Y.A., H.M., T.H, Y.K., K.M., S.O., T.T., Hirotaka Y., H.I., Y.Y., J.M., N.H., K.O., T.O., Hisashi Y., S.N., H.H., Ken I., K.W., N.Y.‐F., and R.H. contributed to data analysis, interpretation, and manuscript preparation. K.Y., Kenta I., Y.A., H.M., T.H., Y.K., K.M., S.O., T.T., Hirotaka Y., H.I., Y.Y., J.M., N.H., K.O., T.O., Hisashi Y., S.N., H.H., Ken I., K.W., N.Y.‐F., and R.H. were involved in participant recruitment and data collection. R.H. supervised the project and was critically involved in the study design, analysis, and interpretation. All authors have approved the final manuscript and agreed to be accountable for all aspects of this study.

## Funding

This study was supported by the Japan Agency for Medical Research and Development (Grant numbers: JP16dk0307060, JP19dk0307083, and JP22dk0307112), JSPS KAKENHI (JP21K17261 and JP23K16273), Health and Labor Sciences Research Grants (Grant numbers: H29‐Seishin‐Ippan‐001 and 19GC1012), Japanese Society of Neuropsychopharmacology, Japanese Society of Mood Disorders, Japanese Society of Clinical Neuropsychopharmacology, and Japanese Society of Psychiatry and Neurology. The funders had no role in the study design, data collection and analysis, decision to publish, or manuscript preparation.

## Ethics Statement

This study was approved by the Ethics Committee of the National Center of Neurology and Psychiatry (approval number: B2022‐004).

## Consent

The authors have nothing to report.

## Conflicts of Interest

The authors declare no conflicts of interest.

## Supporting information


**Table S1:** Satisfaction assessment items for the schizophrenia education program.
**Table S2:** Satisfaction assessment items for the major depressive disorder education program.
**Table S3:** Guidelines for general, schizophrenia, and major depressive disorder groups.

## Data Availability

Data sharing not applicable to this article as no datasets were generated or analysed during the current study.
