# Supplementary material for: Association Between Participant Satisfaction and Self‐Reported Clinical Practice Behaviors in a Nationwide Guideline Education Program
Source: Neuropsychopharmacol Rep. 2026 Jul 14;46(3):e70147. doi: 10.1002/npr2.70147 (PMC13368702; doi:10.1002/npr2.70147)
Supplement: Supplementary file 1 — Table S1: Satisfaction assessment items for the schizophrenia education program. Table S2: Satisfaction assessment items for the major depressive disorder education program. Table S3: Guidelines for general, schizophrenia, and major depressive disorder groups. [file NPR2-46-e70147-s001.docx]

**Supporting Information Table S1. Satisfaction assessment items for the schizophrenia education program**

| 1. How would you rate the content of this course? |
| --- |
| 2. How would you rate your recommendation of this course to a colleague or junior colleague? |
| 3. How would you rate your clinical knowledge regarding the treatment of schizophrenia after attending this course? |
| 4. How would you rate your skills regarding the treatment of schizophrenia after attending this course? |
| 5. How would you rate your confidence in your ability to perform proper treatment of schizophrenia after attending this course? |
| 6. How would you rate your future choice regarding the treatment of schizophrenia according to the guidelines after attending this course? |

This table presents the six items used to assess participants’ satisfaction, including course content, recommendation to colleagues, and self-rated improvements in knowledge, skills, confidence, and guideline-based treatment choices after course completion.

**Supporting Information Table S2. Satisfaction assessment items for the major depressive disorder education program**

| 1. How would you rate the content of this course? |
| --- |
| 2. How would you rate your recommendation of this course to a colleague or junior colleague? |
| 3. How would you rate your clinical knowledge regarding the treatment of major depressive disorder after attending this course? |
| 4. How would you rate your skills regarding the treatment of major depressive disorder after attending this course? |
| 5. How would you rate your confidence in your ability to perform proper treatment of major depressive disorder after attending this course? |
| 6. How would you rate your future choice regarding the treatment of major depressive disorder according to the guidelines after attending this course? |

This table presents the six items used to assess participants’ satisfaction, including course content, recommendation to colleagues, and self-rated improvements in knowledge, skills, confidence, and guideline-based treatment choices after course completion.

**Supporting Information Table S3. Guidelines for general, schizophrenia, and major depressive disorder groups**

| CB-G1: Using treatment guidelines when deciding on the treatment policy in discussion with patients and family. |
| --- |
| CB-G2: Trying to treat patients in accordance with the guidelines if their previous treatments were not in accordance with the guidelines. |
| ***Guideline for Pharmacological Treatment of Schizophrenia 2022*** |
| CB-S1: Choosing antipsychotic monotherapy but not a combination of antipsychotics. |
| CB-S2: Refraining from using psychotropic drugs other than antipsychotics. |
| CB-S3: Providing continuous guidance on the daily administration of antipsychotics. |
| CB-S4: Choosing long-acting injection antipsychotics for patients whose relapse is due to low medication adherence. |
| CB-S5: Defining those with treatment-resistant schizophrenia as patients with schizophrenia who have persistent symptoms despite taking at least two antipsychotics with adequate doses and timing. |
| CB-S6: Choosing treatment with clozapine for patients with treatment-resistant schizophrenia. |
| CB-S7: For recovery from cognitive impairment in schizophrenia, refraining from using anticholinergics. |
| CB-S8: For recovery from cognitive impairment in schizophrenia, refraining from using benzodiazepines. |
| CB-S9: Choosing oral medication for the management of psychomotor agitation, if possible. |
| **New items introduced in *Guideline for Pharmacological Treatment of Schizophrenia 2022***  **(Not present in *Guideline for Pharmacological Therapy of Schizophrenia*)** |
| CB-S10: Reducing doses or switching antipsychotics to prevent extrapyramidal side effects caused by antipsychotic drugs. |
| CB-S11: Refraining from using anticholinergics to prevent akathisia caused by antipsychotic drugs. |
| CB-S12: Refraining from using benzodiazepine receptor agonists to prevent akathisia caused by antipsychotic drugs. |
| CB-S13: Regular monitoring of weight, blood glucose levels, HbA1c, etc., to prevent obesity and diabetes. |
| CB-S14: For insomnia, considering identification of possible causes and providing sleep hygiene instructions. |
| CB-S15: Refraining from prescribing psychotropic medications as pro re nata without clear clinical indications. |
| CB-S16: Choosing treatment with antipsychotic drugs during pregnancy and postpartum (including lactating women). |
| **Old items introduced in *Guideline for Pharmacological Therapy of Schizophrenia***  **(Not present in *Guideline for Pharmacological Treatment of Schizophrenia 2022*)** |
| CB-S17: Choosing medication considering the response to medications in the past for treatment of recurrence or relapse of schizophrenia. |
| CB-S18: Choosing modified electroconvulsive therapy for patients with treatment-resistant schizophrenia. |
| CB-S19: Continuing administration of antipsychotics for at least 1 year for first episode psychosis to prevent relapse. |
| CB-S20: Choosing second-generation antipsychotics to decrease the possibility of extrapyramidal side effects. |
| CB-S21: Ensuring the appropriate dose and timing of pharmacological treatment and the extent of medication adherence in treatment for recurrence or relapse of schizophrenia. |
| ***Treatment Guideline II: Major Depressive Disorder*** |
| CB-D1: Diagnosing depression, including the classification of the severity, based on the DSM-5. |
| CB-D2: In diagnosis, assessing information from any person other than the patient and functional impairment before onset. |
| CB-D3: Focusing on empathic or supportive care and performing fundamental interventions such as psychological education first. |
| CB-D4: When the treatment does not work well, reassessing the diagnosis, pharmacotherapy, and environment management. |
| CB-D5: For mild depression, adding cognitive-behavioral therapy and new-generation antidepressants to fundamental intervention, if necessary. |
| CB-D6: For moderate/severe depression, using antidepressant monotherapy with adequate doses and timing and considering modified electroconvulsive therapy, if necessary. |
| CB-D7: For moderate/severe depression, if antidepressants are effective but not enough, treating with lithium or antipsychotics or T3/T4 as augmentation therapy. |
| CB-D8: Refraining from using long-term administration of anxiolytics. |
| CB-D9: Refraining from using long-term administration of hypnotics. |
| CB-D10: For psychotic depression, using a combination of antidepressants and antipsychotics. |
| CB-D11: For psychotic depression, using modified electroconvulsive therapy. |
| CB-D12: For depression in children and adolescents, providing environment management, psychological education, supportive intervention, and family support before pharmacotherapy. |
| CB-D13: For sleep disorders, first considering differential diagnosis of primary sleep disorders such as obstructive sleep apnea syndrome. |
| CB-D14: For sleep disorders, providing sleep hygiene instructions before pharmacotherapy. |
| **Clinical Guidelines (Not present in this study)** |
| CB-G3: Pharmacotherapy for schizophrenia in your hospital/clinic is in accordance with the guidelines. |
| CB-G4: Recommending pharmacotherapy for schizophrenia to fellow doctors in accordance with the guidelines. |
| CB-G5: Treatment for depression in your hospital/clinic is in accordance with the guidelines. |
| CB-G6: Recommending the treatment for depression to fellow doctors in accordance with the guidelines. |

This table summarizes the clinical behavior items derived from treatment guidelines, including general guideline use, schizophrenia-specific items based on the Guideline for Pharmacological Treatment of Schizophrenia (2022 and earlier versions), and major depressive disorder items based on Treatment Guideline II.

**Abbreviations**: DSM-5, Diagnostic and Statistical Manual of Mental Disorders; T3/T4, triiodothyronine/tetraiodothyronine; CB-G, clinical behaviors related to the general use of guidelines; CB-S, clinical behaviors related to the guidelines for schizophrenia; CB-D, clinical behaviors related to the guidelines for major depressive disorder.

*Guideline for Pharmacological Therapy of Schizophrenia* was utilized as a reference between 2017 and 2021. *Guideline for Pharmacological Treatment of Schizophrenia 2022* was utilized as a reference in 2022 and 2023.
